# A comprehensive evaluation of the effectiveness of a day patient treatment program for eating disorders

**DOI:** 10.1186/2050-2974-2-S1-O24

**Published:** 2014-11-24

**Authors:** Lara Winten, Stephanie Quinton, Suzanne Abraham, Janice Russell, Georgina Luscombe

**Affiliations:** 1 Charles Sturt University, Bathurst, Australia; 2 University of Sydney, Sydney, Australia

## 

Day patient (DP) programs represent a significant development in the treatment of eating disorders, however currently there is a dearth of literature evaluating the effectiveness of DP treatment based upon the targeted goals of treatment. This study aimed to explore the effectiveness and short-term stability of an Australian DP program by examining change across the seven key goals of treatment using a naturalistic pre-post-follow-up study design. The sample included 75 participants meeting criteria for an eating disorder who were admitted to the Northside Clinic DP program. Participants completed measures assessing eating disordered cognitions, readiness to change, quality of life, perpetuating factors, co-morbid factors, body mass index (BMI), and eating disordered behaviours. Fifty-eight patients completed the DP program and 17 dropped-out of treatment. The results demonstrated that there were significant improvements between admission and discharge across all seven targeted areas of treatment, which were maintained at follow-up. Identified predictors of program completion included independent living and attendance travel time of one hour or less. The only identified predictor of good outcome was higher BMI at admission. The results are discussed in the context of other published studies, and potential directions for future research are suggested.

This abstract was presented in the **Service Initiatives** stream of the 2014 ANZAED Conference.

